# Sirt1 coordinates with ERα to regulate autophagy and adiposity

**DOI:** 10.1038/s41420-021-00438-8

**Published:** 2021-03-15

**Authors:** Zhipeng Tao, Limin Shi, Jane Parke, Louise Zheng, Wei Gu, X. Charlie Dong, Dongmin Liu, Zongwei Wang, Aria F. Olumi, Zhiyong Cheng

**Affiliations:** 1 Department of Human Nutrition, Foods, and Exercise, Virginia Tech, Blacksburg, VA 24061 USA; 2 Food Science and Human Nutrition Department, University of Florida, Gainesville, FL 32611 USA; 3 Institute for Cancer Genetics, and Department of Pathology and Cell Biology, and Herbert Irving Comprehensive Cancer Center, College of Physicians and Surgeons, Columbia University, New York, NY 10032 USA; 4 Department of Biochemistry and Molecular Biology, Indiana University School of Medicine, Indianapolis, IN 46202 USA; 5 Department of Surgery, Division of Urology, Beth Israel Deaconess Medical Center, Harvard Medical School, Boston, MA 02115 USA; 6 Present Address: Cutaneous Biology Research Center, Massachusetts General Hospital, Harvard Medical School, Charlestown, MA 02129 USA

**Keywords:** Autophagy, Metabolic disorders

## Abstract

Sex difference in adiposity has long been recognized but the mechanism remains incompletely understood. Previous studies suggested that adiposity was regulated by autophagy in response to energy status change. Here, we show that the energy sensor Sirt1 mediates sex difference in adiposity by regulating autophagy and adipogenesis in partnership with estrogen receptor α (ERα). Autophagy and adipogenesis were suppressed by Sirt1 activation or overexpression, which was associated with reduced sex difference in adiposity. Mechanistically, Sirt1 deacetylated and activated AKT and STAT3, resulting in suppression of autophagy and adipogenesis via mTOR-ULK1 and p55 cascades. ERα induced Sirt1 expression and inhibited autophagy in adipocytes, while silencing Sirt1 reversed the effects of ERα on autophagy and promoted adipogenesis. Moreover, Sirt1 deacetylated ERα, which constituted a positive feedback loop in the regulation of autophagy and adiposity. Our results revealed a new mechanism of Sirt1 regulating autophagy in adipocytes and shed light on sex difference in adiposity.

## Introduction

Autophagy plays a central role in cellular repair, remodeling, development, and homeostasis^1–3^. Autophagy is upregulated during adipocyte differentiation, and inhibition of autophagy suppresses adipogenesis^4–8^. In obese or diabetic individuals, adipose autophagy was shown to be aberrantly activated^9,10^, while targeted suppression of autophagy in the adipose tissue protected against obesity^4,5^. These findings underscore the role of autophagy in adiposity regulation, but the mechanism has not been fully understood and the complexity can be increased by stress conditions (e.g., malnutrition, inflammation, and oxidative stress)^11–13^.

Sirtuin 1 (Sirt1) is an energy sensor that regulates metabolism across tissues^14,15^. Activation or overexpression of Sirt1 improves systemic metabolism and protects against diabetes, obesity, or high-fat diet-induced metabolic damages^16–22^, while dysregulated Sirt1 resulted in phenotypes associated with diabetes, obesity, and aging^23^. In adipocytes, upregulation of Sirt1 enhances lipolysis and attenuates adipogenesis^24^, and ablation of Sirt1 promotes adipocyte differentiation and increases adiposity in mice^25,26^. The roles of Sirt1 in white adipose tissue (WAT) development, maintenance, and remodeling, have been linked to negatively modulating adipogenesis via peroxisome proliferator-activated receptor gamma (PPARγ)^24^, enhancing oxidative phosphorylation via peroxisome proliferator-activated receptor gamma coactivator 1-alpha (PGC-1α)^16–19^, potentiating brown adipose tissue function^22^ or inducing the browning of subcutaneous WAT in response to cold exposure^27^.

Sirt1 was shown to promote or suppress autophagy, partly because of Sirt1 deacetylating autophagy proteins such as Atg5, Atg7, or LC3^28–30^. However, it is unknown whether and how Sirt1 interacts with autophagy in the regulation of adipogenesis and adiposity. In the present study, we investigated the effects of gain and loss of Sirt1 on autophagy and adiposity. We found that the mice with increased expression of Sirt1 showed reduced adiposity, to a greater extent in females than males. Sirt1-induced reduction of adiposity was associated with lower autophagy activity, and the sex difference in adiposity change may be ascribed to the crosstalk between estrogen receptor ERα signaling and Sirt1-autophagy axis. Mechanistically, Sirt1 deacetylated AKT and STAT3, which activated mTOR-ULK1 and STAT3-p55 (p55 subunit of phosphoinositide 3-kinase) signaling pathways, respectively, to mitigate autophagy in adipocytes. ERα and Sirt1 form a positive feedback loop that enhances the effects on autophagy and adiposity. Our study unraveled a new mechanism of Sirt1 regulating adiposity and sex difference through the crosstalk with ERα and autophagy.

## Results

### Sirt1 expression is negatively correlated with autophagy and adipogenesis

**Fig. 1:**
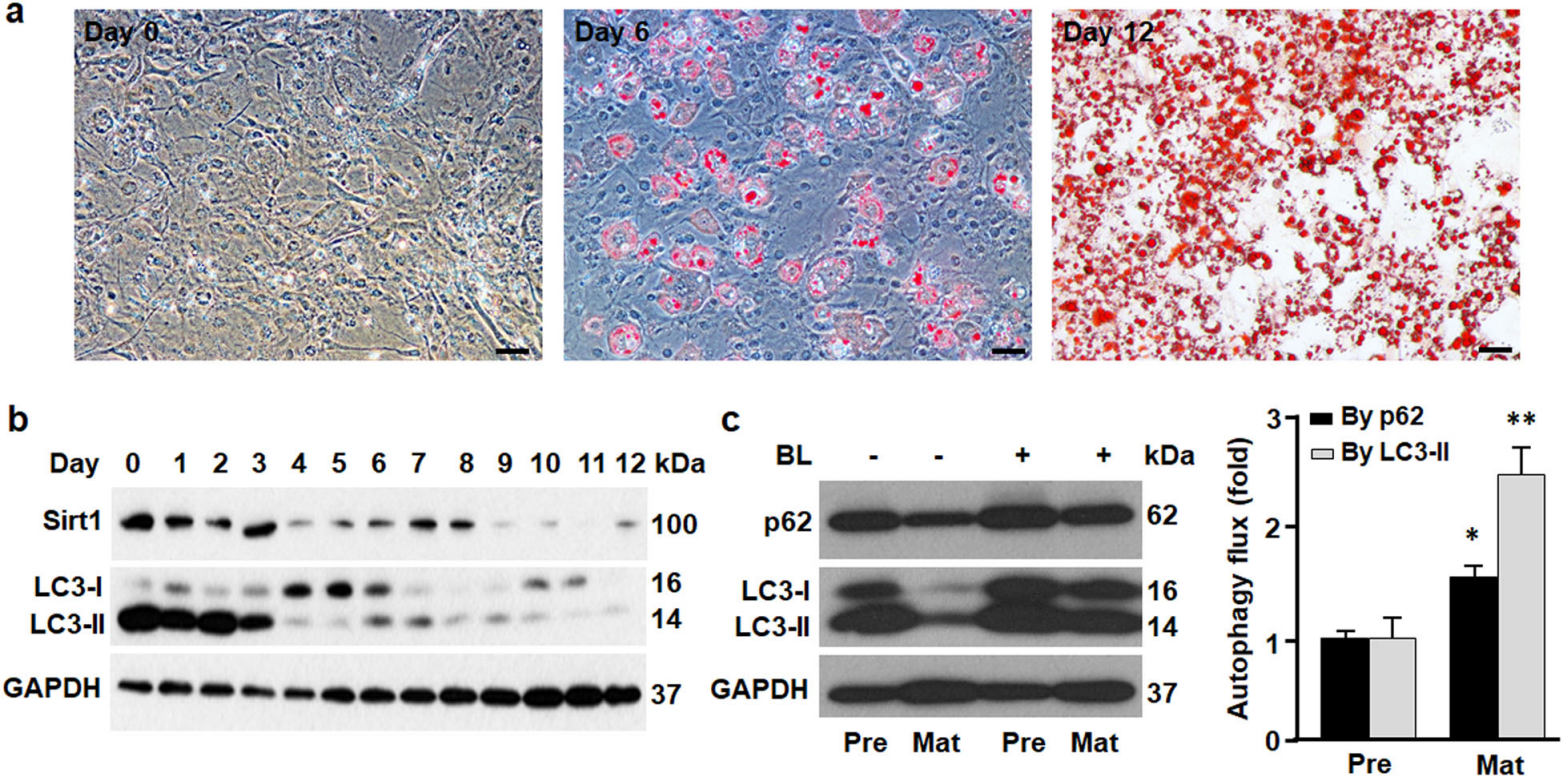
Adipogenesis was associated with downregulation of Sirt1 and activation of autophagy. **a** Oil red O staining of 3T3L1 cells during differentiation, showing the data of day 0 (preadipocyte), day 6 (differentiating adipocyte), and day 12 (differentiated or mature adipocyte). Scale bar, 50 µm. **b** Western blotting analysis of Sirt1 and LC3 proteins during 3T3L1 cell differentiation. GAPDH was probed as a loading control. **c** Autophagy flux was determined by contrasting the rates of removing the substrates LC3-II and p62 by autophagy in the absence and presence of autophagy inhibitors BL (bafilomycin A1 at 0.1 μM and leupeptin at 10 μg/ml). Pre, preadipocyte; Mat, mature adipocyte. *p < 0.05; **p < 0.01. *n* = 8.

During adipogenesis, lipid accumulation was significantly increased (Fig. 1a). Compared with preadipocyte (day 0), Sirt1 expression was drastically reduced in mature adipocyte (day 12), paralleled with significant decrease in LC3-II (Fig. 1b), the substrate that is selectively degraded by lysosomal hydrolase in autolysosome^31,32^. Measurement of autophagy flux (i.e., contrasting the rates of removing substrates LC3-II and p62 by autophagy in the absence and presence of autophagy inhibitors bafilomycin A1 and leupeptin^31^) indicated higher autophagy activity in mature adipocytes than in preadipocytes (Fig. 1c). The inverse correlation between Sirt1 expression and autophagy activity suggests that Sirt1 suppresses autophagy, the cellular remodeling process required for adipocyte differentiation^4–8,33^.

### The effects of gain of Sirt1 on autophagy and adipogenesis

**Fig. 2:**
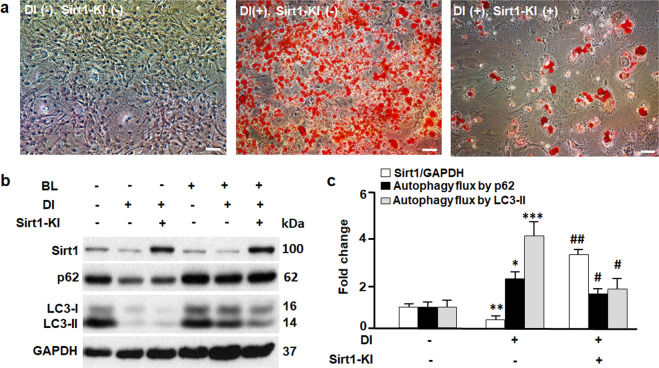
The effects of gain of Sirt1 on autophagy and adipogenesis. **a** Oil red O staining of 3T3L1 cells on day 12, in the absence (−) or presence (+) of differentiation inducer (DI) and Sirt1 knock-in or overexpression (Sirt1-KI). Scale bar, 50 µm. **b** Western blotting analysis of Sirt1, p62, and LC3 proteins. GAPDH was probed as a loading control. Autophagy inhibitors bafilomycin A1 at 0.1 μM and leupeptin at 10 μg/ml (BL) were used to treat the cells and measure the turnover of autophagic substrates p62 and LC3-II (i.e., autophagy flux activities). **c** Densitometric analyses of Sirt1 and autophagy flux. Comparing DI(−)Sirt1-KI(−) with DI(+)Sirt1-KI(−): *p < 0.05; **p < 0.01; ***p < 0.001. Comparing DI(+)Sirt1-KI(−) with DI(+)Sirt1-KI(+): ^#^*p* < 0.05; ^##^*p* < 0.01. *n* = 8.

To examine whether the gain of Sirt1 suppresses autophagy in adipocytes, we overexpressed Sirt1 in 3T3L1 cells using knock-in technique. As shown in Fig. 2a, b, Sirt1 knock-in (Sirt1-KI) significantly increased the expression of Sirt1 protein in 3T3L1 cells, which largely suppressed adipogenesis even in the presence of differentiation inducer (DI). The mitigated adipogenesis was associated with reduced autophagy flux activity (Fig. 2b, c). These findings support the notion that autophagy is required for adipogenesis (Fig. 1)^4–8,33^, and Sirt1 appears to be an autophagy suppressor in adipocytes. In addition, our data, along with the evidence from MEFs, stem cells, and cancer cells^28–30^, suggests that Sirt1 may play tissue- or cell type-dependent roles in autophagy.

### The effects of loss of Sirt1 on autophagy and adipogenesis

**Fig. 3:**
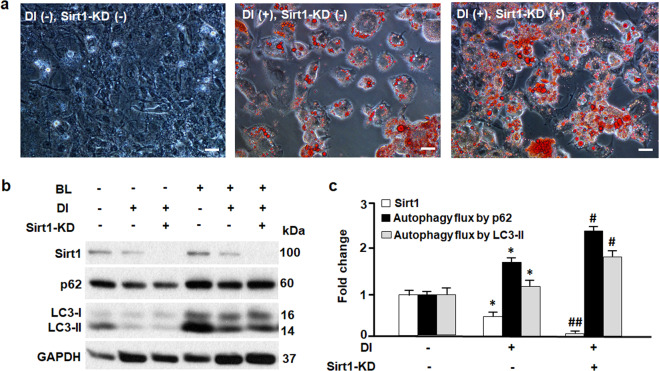
The effects of loss of Sirt1 on autophagy and adipogenesis. **a** Oil red O staining of 3T3L1 cells on day 9, in the absence (−) or presence (+) of differentiation inducer (DI) and Sirt1 knock-down (Sirt1-KD). Scale bar, 100 µm. **b** Western blotting analysis of Sirt1, p62, and LC3 proteins. GAPDH was probed as a loading control. Autophagy inhibitors bafilomycin A1 at 0.1 μM and leupeptin at 10 μg/ml (BL) were used to treat the cells and measure the turnover of autophagic substrates p62 and LC3-II (i.e., autophagy flux activities). **c** Densitometric analyses of Sirt1 and autophagy flux. Comparing DI(−)Sirt1-KD(−) with DI(+)Sirt1-KD(−): *p < 0.05. Comparing DI(+)Sirt1-KD(−) with DI(+)Sirt1-KD(+): ^#^*p* < 0.05; ^##^*p* < 0.01. *n* = 8.

To test whether the loss of Sirt1 may increase autophagy activity and adipogenesis, we used RNA interfering technique to knock down Sirt1 (Sirt1-KD) in 3T3L1 cells (Fig. 3). As shown in Fig. 3a, Sirt1-KD promoted adipocyte differentiation, leading to a greater accumulation of lipid in 3T3L1 cells compared to the 3T3L1 cells incubated with differentiation inducer (DI) alone. Moreover, Sirt1-KD significantly increased autophagy flux activities (Fig. 3b, c). These results underscore Sirt1 as a key suppressor of autophagy during adipogenesis (Figs. 1–3).

### Sirt1 suppresses autophagy via mTOR-ULK1 pathway

**Fig. 4:**
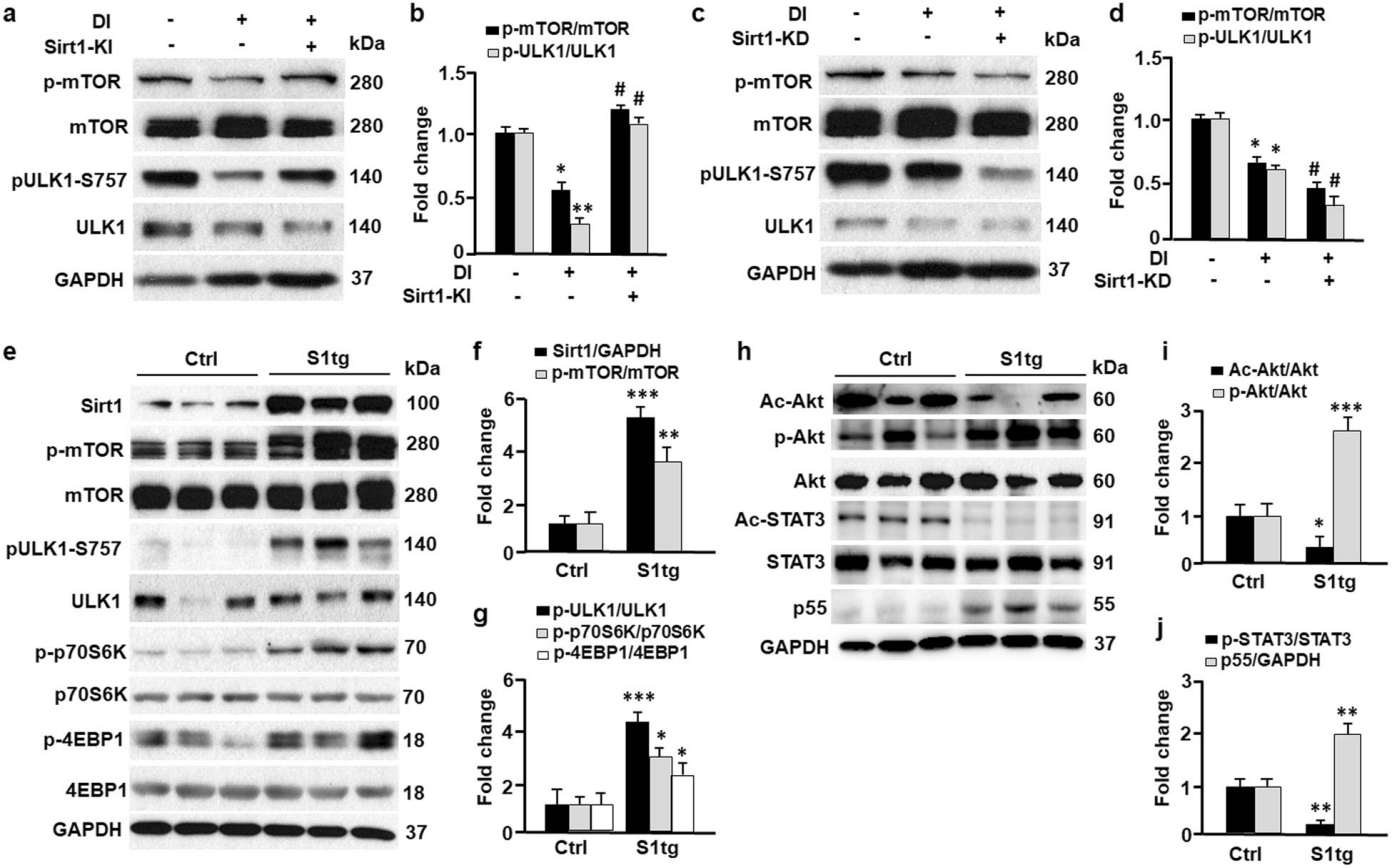
Sirt1 suppressed autophagy by activating AKT-mTOR and STAT3-p55 pathways. **a**, **b** The effects of Sirt1 knock-in (Sirt1-KI) on the mTOR-ULK1 cascade were measured by Western blotting (**a**) and densitometric analyses (**b**). Control and Sirt1-KI 3T3L1 cells were treated with differentiation inducer (DI), and the cells were harvested on day 12 for Western blotting analysis. GAPDH was probed as a loading control. Comparing DI(−)Sirt1-KI(−) with DI(+)Sirt1-KI(−): *p < 0.05; **p < 0.01. Comparing DI(+)Sirt1-KI(−) with DI(+)Sirt1-KI(+): ^#^*p* < 0.05. *n* = 8. **c**, **d** The effects of Sirt1 knock-down (Sirt1-KD) on the mTOR-ULK1 cascade were determined by Western blotting (**c**) and densitometric analyses (**d**). Control and Sirt1-KD 3T3L1 cells were treated with differentiation inducer (DI), and cells were harvested on day 9 for Western blotting analysis. Comparing DI(−)Sirt1-KD(−) with DI(+)Sirt1-KD(−): *p < 0.05. Comparing DI(+)Sirt1-KD(−) with DI(+)Sirt1-KD(+): ^#^*p* < 0.05. *n* = 8. **e**–**g** The effects of Sirt1 on mTOR and downstream targets (ULK1, p70S6K, and 4EBP1) in inguinal adipose tissues were determined by Western blotting (**e**) and densitometric analyses (**f**, **g**). S1tg, Sirt1 transgenic mice; Ctrl, control mice. Comparing Ctrl with S1tg mice: *p < 0.05; **p < 0.01; ***p < 0.001. *n* = 6. **h**–**j** The effects of Sirt1 on protein kinase B (AKT) and signal transducer and activator of transcription 3 (STAT3) in inguinal adipose tissues were determined by Western blotting (**h**) and densitometric analyses (**i**–**j**). Ac-Akt, acetylated Akt; Ac-STAT3, acetylated STAT3. Comparing Ctrl with S1tg mice: *p < 0.05; **p < 0.01; ***p < 0.001. *n* = 6.

Autophagy is a multi-step process that includes initiation, membrane nucleation, expansion, fusion, and degradation^34^. Western blotting analysis suggested that modulation of Sirt1 had marginal effects on beclin 1, Atg5, Atg7, or Atg12-Atg5 conjugate (Fig. 1s), the proteins known to regulate membrane nucleation and expansion^34^. However, overexpression of Sirt1 (Sirt1-KI) in 3T3L1 cells induced inhibitory phosphorylation of ULK1 (p-ULK1-Ser757), the protein that plays a key role in autophagy initiation (Fig. 4a, b). This may account for the suppressed autophagy in Sirt1-KI (Fig. 2). By contrast, knockdown of Sirt1 (Sirt1-KD) drastically attenuated p-ULK1-Ser757 (Fig. 4c, d), which was associated with deactivation of the serine/threonine protein kinase mTOR (i.e., dephosphorylation of mTOR at Ser2448)^35,36^. Consistently, Sirt1-KI significantly upregulated p-mTOR-Ser2448, known to enhance mTOR kinase activity and suppress autophagy by phosphorylating ULK1 at Ser757 (Fig. 4a, b)^37^. To confirm the effects of Sirt1 on mTOR activity in vivo, we studied the adipose tissues from Sirt1 transgenic (S1tg) mice (Fig. 4e–g)^20^. Overexpression of Sirt1 activates mTOR by significantly increasing the phosphorylation level at Ser2448 (p-mTOR-Ser2448), which was associated with an elevation in mTOR-mediated inhibitory phosphorylation on ULK1 (p-ULK1-Ser757). As the downstream targets of mTOR, the proteins p70S6K and 4EBP1 exhibited significantly higher phosphorylation levels in S1tg mice compared with the control mice (Fig. 4e–g)^36,38^. Therefore, Sirt1 activates the mTOR-ULK1 cascade to dampen autophagy.

### Sirt1 deacetylates and activates AKT and STAT3

Previous studies showed that Sirt1 might regulate autophagy due to deacetylation of Atg5, Atg7, or LC3 in cancer cells, MEFs, germ cells, and stem cells^28–30,39^. Using immunoprecipitation and immunoblotting analyses, we pulled down acetylated proteins and probe Atg5, Atg7, and LC3 in adipose tissues from control and S1tg mice. Unexpectedly, S1tg and the control mice showed comparable acetylation in Atg5, Atg7, and LC3 (Fig. 2s). However, overexpression of Sirt1 drastically deacetylated protein kinase B (Akt) and signal transducer and activator of transcription 3 (STAT3) in S1tg mice (Fig. 4h–j). Deacetylation of Akt was shown to enhance its phosphorylation and kinase activity in phosphorylating mTOR^40^, and deacetylation of STAT3 increases its transcription factor activity^41–43^. Indeed, Akt phosphorylation and p55 expression were markedly upregulated in S1tg mice in comparison to the control mice (Fig. 4h–j). Consistently, phosphorylation of mTOR and its downstream target proteins p70S6K and 4EBP1 were induced in S1tg mice (Fig. 4e–g), supporting the notion that Akt activates mTOR^44–47^. Moreover, the activation of STAT3-p55 cascade by Sirt1 may serve as an additional mechanism of the mitigated autophagy because p55 was depicted as an inhibitor of autophagy (Fig. 4h, j)^42^.

### Sirt1 reduces sex difference in adiposity

**Fig. 5:**
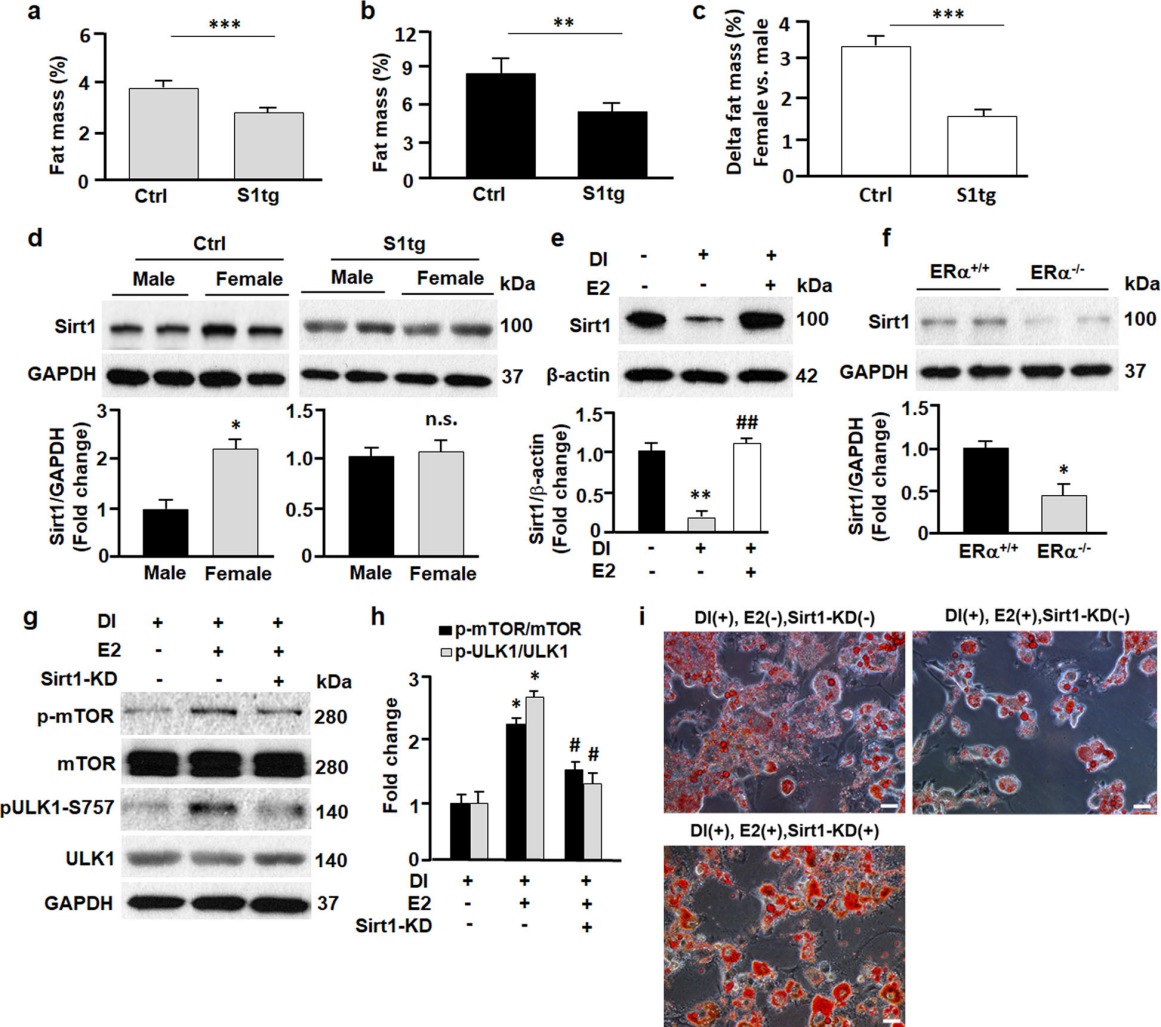
Sirt1 regulated sex difference in adiposity. **a**, **b** Fat mass in male (**a**) and female (**b**) mice. **p < 0.01; ***p < 0.001; *n* = 6–10. **c** Sex difference (females vs males) in adiposity was greater in Ctrl mice compared to S1tg mice. ***p < 0.001; *n* = 6–10. **d** Sirt1 expression in inguinal adipose tissues was analyzed by Western blotting and densitometric analyses. *p < 0.05; n.s., not significant; *n* = 6. **e** The effects of estradiol (E2, 0.1 μM) on Sirt1 in 3T3L1 cells were analyzed by Western blotting and densitometric analyses. β-actin was probed as a loading control. **p < 0.01 by comparing DI(−)E2(−) with DI(+)E2(−). ^##^*p* < 0.01 by comparing DI(+)E2(−) with DI(+)E2(+). *n* = 8. **f** The effects of estrogen receptor α knockout (ERα^−/−^) on Sirt1 in inguinal adipose tissues were analyzed by Western blotting and densitometric analyses. GAPDH was probed as a loading control. *p < 0.05 by comparing estrogen receptor α knockout (ERα^−^^/^^−^) mice with wild type (ERα^+/+^) mice. *n* = 8. **g** The effects of E2 (0.1 μM) and Sirt1 knockdown (Sirt1-KD), alone or combined, on mTOR-ULK1 cascade (day 9). Western blotting (**g**) and densitometric analyses (**h**) were employed to determine the effects. *p < 0.05 by comparing DI(+)E2(−)Sirt1-KD(−) with DI(+)E2(+)Sirt1-KD(−). ^#^*p* < 0.05 by comparing DI(+)E2(+)Sirt1-KD(−) with DI(+)E2(+)Sirt1-KD(+). *n* = 8. **i** The effects of E2 and Sirt1-KD, alone or combined, on adipogenesis (day 9). Scale bar, 100 µm.

To examine how the Sirt1-autophagy axis affects adiposity in vivo, we measured the fat mass of S1tg mice and control mice (Fig. 5a, b). S1tg mice exhibited significantly lower fat mass compared with the control mice, in line with Sirt1 suppressing autophagy and adipogenesis in vitro (Figs. 1–3) and Sirt1 activation preventing adipose expansion in mice^16,17^. Intriguingly, the sex difference in S1tg mice was attenuated drastically in comparison to control mice (Fig. 5c). Western blotting analysis revealed that in control mice, females had a higher expression of Sirt1 than in males, while the difference was largely abolished by Sirt1 overexpression in S1tg mice (Fig. 5d). These findings suggested the Sirt1 might crosstalk with sex hormone signaling, particularly estrogen receptor ERα signaling, because ERα has been shown to regulate autophagy and adiposity^7,48,49^. Indeed, treatment of 3T3L1 cells with estradiol (E2) markedly induced Sirt1 expression (Fig. 5e), while knockout of ERα attenuated Sirt1 expression (Fig. 5f), underscoring Sirt1 as a downstream target of ERα. When adipocytes were treated with E2, it increased phosphorylation of mTOR and ULK1 (Fig. 5g, h), the signaling pathway known to mitigate the initiation of autophagy (Figs. 1–4)^7,34^. In line with autophagy required for adipogenesis, E2 treatment substantially inhibited adipocyte differentiation (Fig. 5i). However, knockdown of Sirt1 (Sirt1-KD) abolished E2-induced effects on the mTOR-ULK1 cascade and adipogenesis, further confirming that E2/ERα signaling regulates autophagy and adipogenesis by inducing Sirt1 expression (Fig. 5g–i). Therefore, the reduced sex difference in adiposity in S1tg mice may be explained at least in part by the fact that Sirt1 overexpression overrides the effects of E2/ERα.

### Sirt1 deacetylates ERα and reduces adiposity

**Fig. 6:**
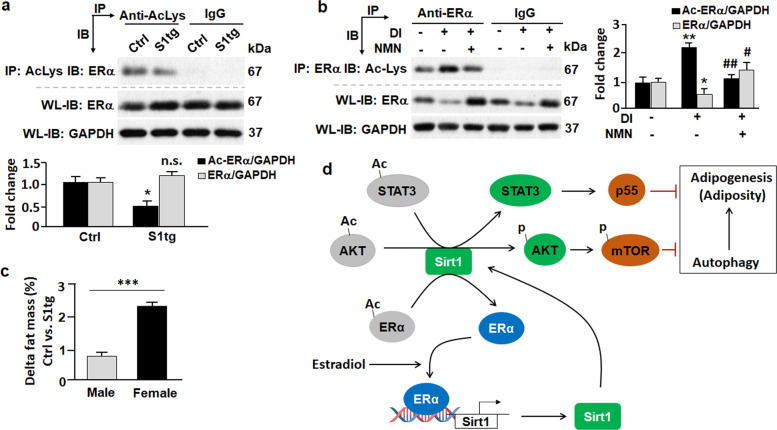
Sirt1-induced deacetylation of ERα and reduction of adiposity in mice. **a** Effects of Sirt1 overexpression on ERα acetylation in mice. Upper, acetylated ERα was analyzed by immunoprecipitation (IP) with an anti-acetylated lysine (anti-acLys) antibody that pulled down acetylated proteins, followed by immunoblot (IB) with an anti-ERα antibody. Normal rabbit IgG (non-specific IgG) was used as negative control. Middle, whole lysates (WL) were analyzed by IB with anti-ERα and anti-GAPDH antibodies. Lower, densitometric analyses were used to quantify acetylated and total ERα in control (Ctrl) and Sirt1 transgenic (S1tg) mice. *p < 0.05; *n* = 8. **b** Effects of Sirt1 activator nicotinamide mononucleotide (NMN, 100 µM) on ERα acetylation in 3T3L1 cells. Left-upper, acetylated ERα was analyzed by IP with an anti-ERα antibody that pulled down ERα protein, followed by IB with an anti-acetylated lysine (anti-acLys) antibody. Normal rabbit IgG (non-specific IgG) was used as the negative control. Left-lower, whole lysates (WL) were analyzed by IB with anti-ERα and anti-GAPDH antibodies. Right, densitometric analyses were used to quantify acetylated and total ERα in 3T3L1 cells. DI, differentiation inducer. *p < 0.05 and **p < 0.01 by comparing DI(−) NMN(−) with DI(+) NMN(−). ^#^*p* < 0.05 and ^##^*p* < 0.01 by comparing DI(+) NMN(−) with DI(+) NMN(+). *n* = 8. **c** Sirt1-induced reduction of adiposity was greater in female than in male mice. ***p < 0.001; *n* = 6–10. **d** A schematic view of Sirt1 coordinating with ERα to regulate autophagy and adipogenesis (adiposity).

Given the nature of Sirt1 as a deacetylase, we asked the question whether adipose ERα is deacetylated by Sirt1. Intriguingly, overexpression of Sirt1 significantly reduced acetylation level of ERα in S1tg mice in comparison to the control mice (Fig. 6a). Total ERα protein level was higher in S1tg mice than in the control mice, but the difference was not statistically significant (Fig. 6a). In mature 3T3L1 adipocytes (DI+/NMN−), acetylation of ERα increased by over twofold (*p* < 0.01) in comparison to preadipocytes (DI−/NMN−), as shown in Fig. 6b. Total ERα protein level was reduced in mature 3T3L1 adipocytes, consistent with the notion that suppression of ERα signaling promotes adipogenesis^7^. However, when 3T3L1 cells were treated with nicotinamide mononucleotide (NMN, 100 µM), a Sirt1 activator that promotes biosynthesis of NAD^+^^15,50,51^, it significantly reduced acetylation level of ERα (Fig. 6b). These in vitro and in vivo data confirm that ERα is deacetylated by Sirt1, suggesting that Sirt1 forms a positive feedback loop and promotes E2/ERα signaling cascade in the regulation of adiposity. In line with this, Sirt1 overexpression in males reduced adiposity by 0.7% (Fig. 6c); however, Sirt1 overexpression in females (known to have stronger E2/ERα signaling^52,53^) resulted in greater reduction of adiposity, i.e., by 2.3% (*p* < 0.001 vs. 0.7% in the males). Thus, our data support the model where E2/ERα signaling induces Sirt1-mediated suppression of autophagy and adipogenesis, and Sirt1 forms a positive feedback loop to enhance E2/ERα signaling in the regulation of adiposity (Fig. 6d).

## Discussion

The mechanism of sex difference in adiposity is complex and remains incompletely understood^54,55^. Previous research has implicated both sex chromosome and hormones in adipose development and growth^7,54,55^. In this study, we identified an E2/ERα-Sirt1 axis that regulates autophagy and sex difference in adiposity. Activation of E2/ERα signaling induced Sirt1 expression (Fig. 5e, f), presumably through transactivation by binding to the promoter of Sirt1 gene and increasing its transcription^56^. Overexpression of Sirt1 phenocopied the effects of E2/ERα signaling, during which mTOR-ULK1 cascade was activated and dampened autophagy and adipogenesis (Figs. 2, 4 and 5)^7^. Consistently, silencing Sirt1 abolished the effects of E2/ERα signaling on autophagy and adipogenesis (Figs. 3 and 5). In line with E2/ERα signal differing in males and females^52,53^, Sirt1 expression was higher in females vs. males in the control mice (Fig. 5d). However, the sex difference in Sirt1 expression was normalized in S1tg mice, and the sex differences in adiposity was significantly reduced in comparison to control mice (*p* < 0.001, Fig. 5c).

Additional mechanism of Sirt1-induced suppression of autophagy includes the STAT3-p55 pathway. STAT3 was shown to disrupt lysosomal function^57^.The p55 subunit of phosphoinositide 3-kinase acts as a direct inhibitor of autophagy by blocking ability of p85 in autophagy initiation^42^. We found that Sirt1 deacetylated and activated STAT3, which drastically induced p55 expression (Fig. 4h, j). Activation of STAT3 and resultant induction of p55 accounts at least in part for the mitigated autophagy in cells or animals overexpressing Sirt1. Of note, Sirt1 appears to constitute a positive feedback with ERα signaling via deacetylation and possibly transcriptional regulation^58^, which warrants further investigation in the future (Fig. 6d). Altogether, our data suggest that Sirt1 serves as a suppressor of autophagy by activating AKT-mTOR and STAT3-p55 cascades, which mediates sex difference in adiposity via crosstalk with E2/ERα signaling.

Our study is the first to document the role of Sirt1 in regulating autophagy in adipose tissue. Sirt1 appeared to act on the initiation of autophagy via inhibitory phosphorylation of ULK1 by mTOR. Notably, Sirt1 overexpression resulted in marginal changes in phosphorylation of mTOR and ULK1 in skeletal muscle (data not shown), suggesting that Sirt1 plays tissue- or cell type-dependent roles in autophagy. Indeed, Sirt1 induces autophagy by deacetylating and activating Atg5, Atg7, or LC3, but this function was absent in certain cell types^28–30,39^. For instance, deacetylation of LC3 and Atg7 leads to activation of autophagy in spermatids; nevertheless, knockout of Sirt1 attenuated autophagy and spermiogenesis in germ cells but not in steroidogenic cells^39^. It is, therefore, of interest for future studies to explore the molecular and cellular factors that determine the cell type-dependent roles for Sirt1 in the regulation of autophagy.

## Materials and methods

### Mice

The S1tg and control mice were bred and housed as described previously^7,20^. The ERαKO (ERα^−/−^) and control mice (ERα^+/+^) were obtained by breeding heterozygous males to females^7^. All the mice were housed in plastic cages on a 12-h light–dark photocycle and with free access to water and regular chow diet^7,59^. At the age of 6–8 weeks, the S1tg and control mice were weighed, and fat mass was measured with a Bruker Minispec LF90 NMR Analyzer (Bruker Optics, Billerica, MA, USA), and then euthanized for tissue collection. ERαKO and control mice were sacrificed for tissue collection at the age of 12–16 weeks. The inguinal WAT pads were collected and weighed quickly and snap freezing in liquid nitrogen. Power calculation suggested that 6 mice per group were required to obtain statistically meaningful data. Mice were randomly grouped for each genotype. Animal use procedures followed the National Institutes of Health guidelines and were approved by the Institutional Animal Care and Use Committees at University of Florida and Virginia Tech.

### 3T3L1 cell culture, differentiation, and treatment

3T3L1 preadipocytes (ATCC CL-173, Manassas, VA, USA) were cultured in basal media (DMEM media containing 10% FBS, 100 units/ml penicillin and 100 μg/ml streptomycin (1× P/S)), at 37 °C in a humidified atmosphere of 5% CO_2_^31,60^. The media were replaced every 2 days until the cells became confluent (day 0), and after 2 more days (day 2) the media were changed to differentiation medium I (DMEM with 10% FBS, P/S (1×), IBMX (0.5 mM), dexamethasone (1 μM), insulin (1 μg/ml), and rosiglitazone (2 μM)). At the end of day 4, the media were changed to differentiation medium II (DMEM with 10% FBS, P/S (1×), and insulin (1 μg/ml)). At the end of day 6, the media were changed to basal media and the cells were maintained until day 12. Preadipocytes without differentiation induction were maintained in basal media and supplied with fresh medium every 2 days till day 12. Resveratrol (RSV) at the concentrations of 0–50 μM, NMN at concentration of 100 μM, and β-estradiol (E2) of 0.1 μM, were used to treat cells as indicated. Other chemicals were used at the concentrations established previously, including bafilomycin A1 (4 nM), and leupeptin (0.4 ng/ml)^6^, to treat the cells during differentiation.

### Cell transfection

After cells reached 80–90% confluent, overexpression (Sirt1-KI) or knockdown (Sirt1-KD) of Sirt1 was achieved by incubating the cells with adenoviruses carrying GFP (control) and Sirt1 coding sequences, or adenoviruses carrying GFP (GCATCAAGGTGAACTTCAAGA, control) and Sirt1 (GCACCGATCCTCGAACAATTC) shRNA sequences, at 100 multiplicity of infection^61^. After 2 days, cell differentiation was induced as described above and cells were analyzed or harvested on day 9 or day 12 as indicated.

### Oil red O staining

The Oil Red O working solution was freshly prepared by mixing 0.35% stock solution with dH_2_O (6:4) and filtered, and the staining was conducted as described^6,33,60^. After the media were removed, the cells were washed once with cold phosphate buffered saline and fixed in 4% formaldehyde at room temperature for 10 min. The cells were then washed with dH_2_O and air dried. Oil Red O working solution was added to start the staining at room temperature for 30 min. The stained cells were washed with dH_2_O for 4 times before the images were captured with a Nikon ECLIPSE Ti Inverted Microscope (Melville, NY, USA).

### Autophagy flux assay

Preadipocytes and mature adipocytes were treated with bafilomycin A1 (inhibitor of autophagosome acidification, at 0.1 μM) plus leupeptin (the inhibitor of lysosomal proteases, at 10 μg/ml) for 4 h. The cells were then harvested to prepare cell lysates as previously described^6,33,60^. The turnover of LC3-II or p62 protein, i.e., the autophagic removal of the substrate, was measured by Western blotting and image analysis to assess autophagy flux^6,31,62^.

### Immunoprecipitation

Immunoprecipitation was conducted to purify and enrich acetylated ERα to ensure the specificity and sensitivity of detection as described previously:^15,63^ anti-acetyl Lysine (ab190479) antibody and protein A/G Sepharose (ab193262) from abcam (Cambridge, MA, USA); ERα (04-820) antibody from EMD Millipore (Billerica, MA, USA); normal rabbit IgG (#2729) from Cell Signaling Technology (Beverly, MA, USA).

### Western blotting

Tissue and cell lysates were prepared with PLC lysis buffer (30 mM Hepes, pH 7.5, 150 mM NaCl, 10% glycerol, 1% Triton X-100, 1.5 mM MgCl_2_, 1 mM EGTA, 10 mM NaPPi, 100 mM NaF, 1 mM Na_3_VO_4_) supplemented with protease inhibitor cocktail (Roche), and 1 mM PMSF^6,31,60^. Total protein concentrations of the lysates were determined using a DC protein assay kits (Bio-Rad). Antibody (catalog number) information: GAPDH (MA5-15738) and β-actin (MA5-15739) antibodies were purchased from Pierce (Rockford, IL, USA); p-mTOR (Ser2448) antibody (5536s) and p-ULK1(Ser757) antibody (14202s) from Cell Signaling Technology (Beverly, MA, USA); ERα (04-820) and Sirt1 (07-131) antibodies from EMD Millipore (Billerica, MA, USA); p62 (ab56416) antibody from abcam (Cambridge, MA, USA); and LC3B antibody (L7543) from Sigma (Billerica, MA, USA).

### Statistical analysis

Measurements were duplicated or triplicated, with 6–10 mice included in each group. Data were presented as mean ± SD. Unless the use of female mice were specified, the animal studies were conducted on males. Differences between groups and treatments were validated by one-way analysis of variance or a two-sided *t-*test. A value of *p* < 0.05 was considered statistically significant.

## Supplementary information

Supplemental Figure Legends

Suppl Fig 1

Suppl Fig 2
