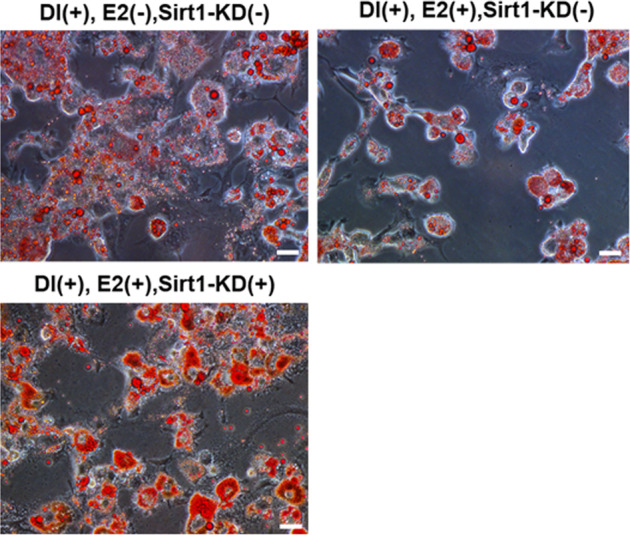# Correction: Sirt1 coordinates with ERα to regulate autophagy and adiposity

**DOI:** 10.1038/s41420-023-01471-5

**Published:** 2023-06-23

**Authors:** Zhipeng Tao, Limin Shi, Jane Parke, Louise Zheng, Wei Gu, X. Charlie Dong, Dongmin Liu, Zongwei Wang, Aria F. Olumi, Zhiyong Cheng

**Affiliations:** 1 Department of Human Nutrition, Foods, and Exercise, Virginia Tech, Blacksburg, VA 24061 USA; 2 Food Science and Human Nutrition Department, University of Florida, Gainesville, FL 32611 USA; 3 Institute for Cancer Genetics, and Department of Pathology and Cell Biology, and Herbert Irving Comprehensive Cancer Center, College of Physicians and Surgeons, Columbia University, New York, NY 10032 USA; 4 Department of Biochemistry and Molecular Biology, Indiana University School of Medicine, Indianapolis, IN 46202 USA; 5 Department of Surgery, Division of Urology, Beth Israel Deaconess Medical Center, Harvard Medical School, Boston, MA 02115 USA; 6 Present Address: Cutaneous Biology Research Center, Massachusetts General Hospital, Harvard Medical School, Charlestown, MA 02129 USA

**Keywords:** Autophagy, Metabolic disorders

Correction to: *Cell Death Discovery* 10.1038/s41420-021-00438-8, published online 15 March 2021

The original version of this article contained an error. The authors noted an oversight in uploading and assembling images for the Figure, which led to the lower panel of Fig. 5i being assigned to an incorrect image in the version of this paper initially published. The revised Fig. 5i that contains the correct image for the treatment of [DI(+), E2(+),Sirt1-KD(+)] is shown below. This change does not affect the conclusions in the paper. The authors apologize for any confusion that this error may have caused.