# Supplementary material for: Sirt1 coordinates with ERα to regulate autophagy and adiposity
Source: Cell Death Discov. 2021 Mar 15;7:53. doi: 10.1038/s41420-021-00438-8 (PMC7960718; doi:10.1038/s41420-021-00438-8)
Supplement: Supplementary file 1 — Supplemental Figure Legends [file 41420_2021_438_MOESM1_ESM.docx]

**Supplemental data**

**Figure 1s Western blotting analysis of autophagy regulating proteins.** **a** Sirt1 knockdown (Sirt1-KD) had marginal effects on beclin 1, Atg5, and Atg7 in 3T3L1 cells on day 12. DI, differentiation inducer. n.s., not significant; n = 8. **b** The effect of Sirt1 overexpression on autophagy proteins in inguinal adipose tissue from Sirt1 transgenic (S1tg) mice. Compared to the control (Ctrl) mice, S1tg mice showed marginal changes in beclin 1, Atg5, Atg7, and Atg12-Atg5 conjugate. GAPDH was probed as a loading control. n.s., not significant; n = 6.

**Figure 2s** **Effects of Sirt1 on acetylation of Atg5, Atg7, and LC3.** Inguinal adipose tissue lysates were used for immunoprecipitation (IP) and immunoblot (IB) to probe acetylated Atg5, Atg7, and LC3. Compared to the control (Ctrl) mice, S1tg mice showed no significant change in acetylated Atg5, Atg7, or LC3. n.s., not significant; n = 6.
