# Supplementary figures and images for: Sirt1 coordinates with ERα to regulate autophagy and adiposity
Source: Cell Death Discov. 2021 Mar 15;7:53. doi: 10.1038/s41420-021-00438-8 (PMC7960718; doi:10.1038/s41420-021-00438-8)

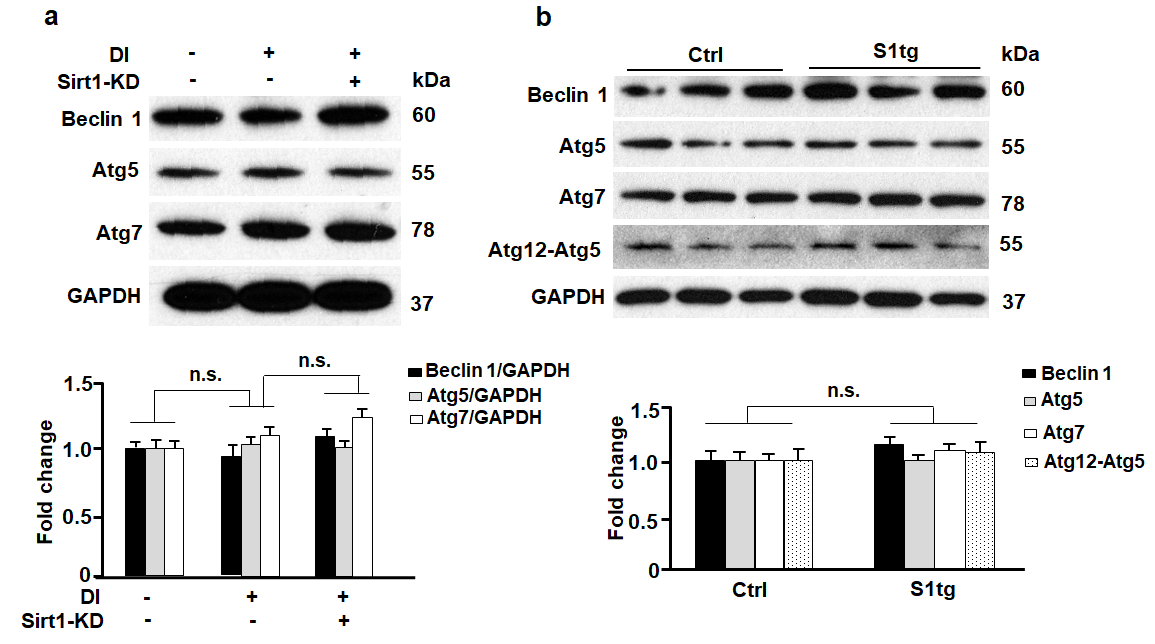

Supplement: Supplementary file 2 — Suppl Fig 1 [file 41420_2021_438_MOESM2_ESM.tif]

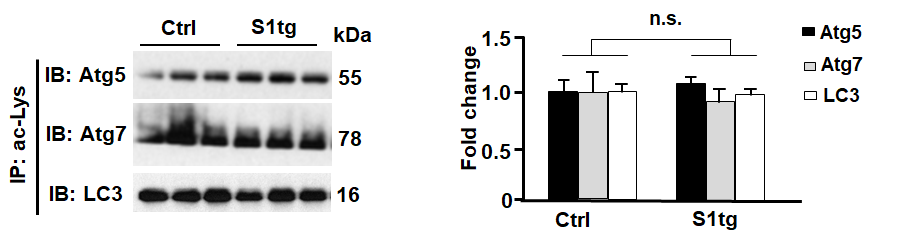

Supplement: Supplementary file 3 — Suppl Fig 2 [file 41420_2021_438_MOESM3_ESM.tif]
